# A new species of *Xylotrechus* Chevrolat from China (Coleoptera, Cerambycidae)

**DOI:** 10.3897/zookeys.722.13265

**Published:** 2017-12-13

**Authors:** Shulin Yang, Weicheng Yang

**Affiliations:** 1 School of Life Sciences, Guizhou Normal University, Guiyang, Guizhou, China; 2 Research Center for Karst Caverns, Guizhou Normal University, Guiyang, Guizhou, China

**Keywords:** Guizhou, Leigongshan Nature Reserve, longhorn beetles, taxonomy

## Abstract

*Xylotrechus tristisfacies* **sp. n.** (Coleoptera, Cerambycidae, Cerambycinae, Clytini) from China is described and illustrated. Characters distinguishing the new species from its close relatives, which possess an entirely black or dark brown pronotum with a black median stripe on the disc and dense yellowish to gray pubescent elytra with black or brown spots or bands, are presented.

## Introduction

*Xylotrechus* Chevrolat, 1860 is a genus of the tribe Clytini Mulsant, 1839 characterized by one or more vertical or branching carina on the forehead. There are over 210 species and subspecies described worldwide. Among them, 125 species and subspecies are recorded in the Palaearctic region and 70 of them are distributed in China (Tavakilian and Chevillotte 2016, Danilevsky 2017). Specimens representing a new species of *Xylotrechus* were collected in a survey for the Leigongshan National Nature Reserve within Leishan County, Guizhou Province of China. The type material is preserved in the School of Life Sciences, Guizhou Normal University, Guiyang, China (GZNULS).

## Taxonomy

### Xylotrechus tristisfacies sp. n.

Taxon classificationAnimaliaColeopteraCerambycidae

http://zoobank.org/E124F1E2-D4D6-499D-9185-CEB6F1B9BF95

Figure 1

#### Type material.

Holotype ♂: Leigongshan National Nature Reserve, Leishan County, Guizhou Province, CHINA, 2016.VIII.31, 26°22'25"N, 108°11'58"E, leg. S. Yang (GZNULS). Paratypes: two ♀, same data as holotype except 2016.IX.21 (GZNULS); Four ♀, same location as holotype, 2017.X.7, leg. Yaokui Yang and Gugangzu Yang (GZNULS).

**Figure 1.**
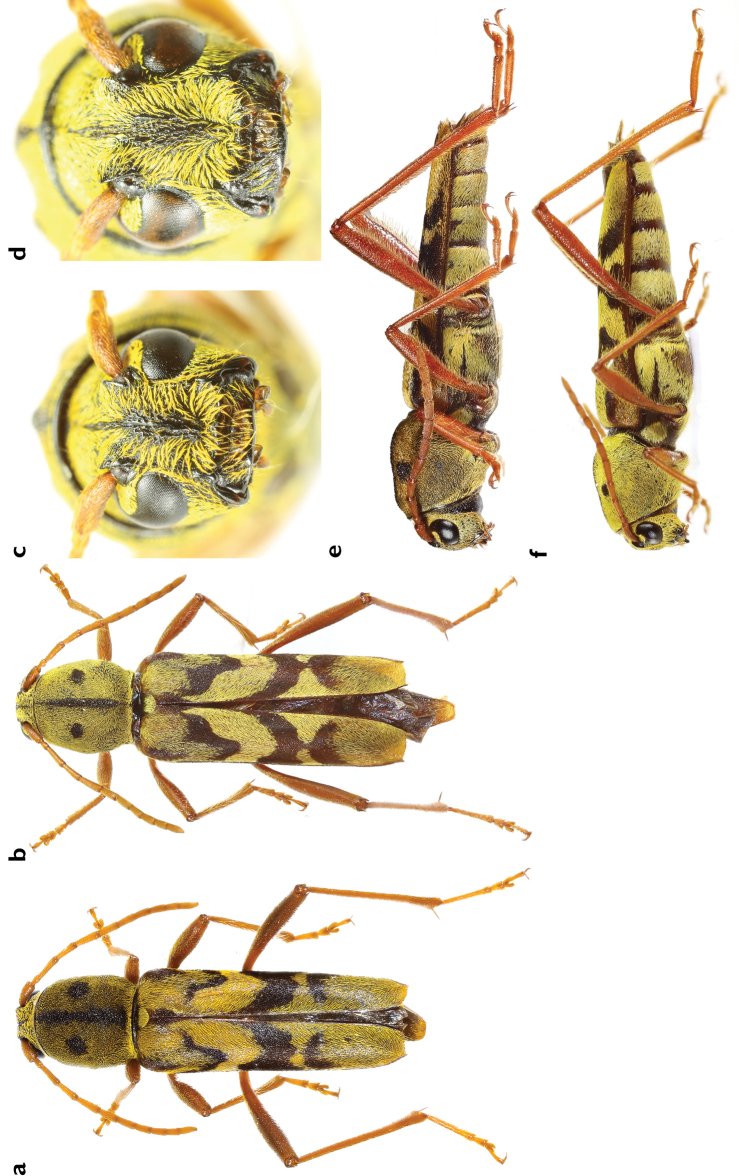
*Xylotrechus tristisfacies* sp. n., Male. **a** dorsal view **c** front view **e** lateral view Female. **b** dorsal view **d** front view **f** lateral view.

#### Differential diagnosis.

Characters of *Xylotrechus tristisfacies* sp. n. place it into the subgenus Xylotrechus, especially into the species group whose elytra have black integuments clothed with dense yellow pubescence broken by black markings and the pronotum shows a black median stripe. This species group contains *X. polyzonus* (Fairmaire, 1888), *X. multisignatus* Pic, 1915, *X. incurvatus incurvatus* (Chevrolat, 1863), and *X. incurvatus contortus* Gahan, 1906 (Gressitt 1951, key couplets 25–27 for genus *Xylotrechus*). Though *X. atrolineatus* Pic, 1917 was not included in Gressitt (1951), it is close to *X. incurvatus contortus* and could be included into this group. Subsequently described species, such as *X. vinnulus* Holzschuh, 1993 and *X. securus* Holzschuh, 2009, could also be included into this group.

Similar to the new species, *X. bilyi* (Holzschuh, 2003), *X. savioi* (Pic, 1935), *X. securus* (Holzschuh, 2009), *X. daoi* (Gressitt & Rondon, 1970) and *X. klapperichi* (Gressitt, 1951) also have three black bands on each elytron. However, the new species differs from *X. bilyi*, *X. savioi*, and *X. daoi* in pronotal marking pattern, i.e., one median black ridge and one black round marking on each side of the ridge. In contrast, *X. bilyi* has one triangular marking in the middle and one on each side, *X. savioi* has three round black dots, one in the middle and one on each side, and *X. daoi* has one round black spot on each side and no median ridge.

The new species differs from *X. klapperichi*, which it resembles in general habitus, in the pronotum obviously narrower than elytra (pronotal width subequal to the elytral width in *X. klapperichi*), distinct black humeral markings, short lengthwise post-scutellar black markings (which *X. klapperichi* does not have), post-median elytral stripe more curving towards apex, and rather dense yellow pubescence with a caret shaped black marking on the apical third (not entirely pitch-black on the apical fourth as in *X. klapperichi*).

*Xylotrechus securus*, which also has one median black ridge and two round markings on the pronotum, was described from Laos (Holzschuh 2009) and it is the closest congener of *X. tristisfacies*. The elytral band pattern can differentiate these two species. The sutural end of the first elytral black band, starting from approximately the basal sixth, turns laterally at basal third in *X. tristisfacies*. The first elytral band of *X. securus* starts about basal fourth from the sutural end to nearly half of the elytral length to the lateral end. The 2^nd^ elytral band of *X. tristisfacies* is wider than that of *X. securus*.

#### Description.

Male (Fig. 1a, c, e): Body cylindrical; integument dark reddish brown to black, clothed with yellow and black pubescence forming black stripes and patches on pronotum and elytra. Length: 13.9 mm. Face and genae clothed with yellow pubescence, striated smooth black stripe, which narrow at bottom and gradually wide towards antennal insertions in the middle of frons; vertex densely punctured, also clothed with yellow pubescence except a narrow median black line. Antennae 11-segmented, short, not reaching hind femurs; 6^th^ antennomere slightly surpassing the humeri; reddish-brown clothed with yellow hairs; these hairs longer on antennomeres I–V, especially on inner side of antennomeres II–V, suberect and forming small hair clusters; 3^rd^ antennomere longer than scape, 4^th^ and other antennomeres; 4^th^ and 6^th^ antennomeres subequal in length and shorter than 5^th^; 7^th^ antennomere subequal to 6^th^; 8^th^ through 11^th^ antennomeres subequal and shorter than 7^th^. Prothorax longer than wide with nearly parallel sides, narrower than elytra; surface of disc densely punctured, clothed with yellow hairs except a median ridge and two round patches on the disc; ridge and patches rugulose and slightly raised. Elytra subparallel-sided, reddish brown, approximately 2.6 times longer than width at humeri; apices obliquely sub-truncate with outer angle minutely acute; mostly covered with short, dense yellow pubescence, thinner at basal and apical third; two lengthwise short black stripes at basal of each elytron, one at humerus and one at suture after scutellum; three transverse black bands on each elytron: 1^st^ band V-shaped (outer half of the band shorter), from basal sixth to basal third, sutural apex of the band extending transversely towards suture but not reaching suture; 2^nd^ band wider, curving towards apex to the middle of elytron, narrowing down obliquely forward and reaching suture; 3^rd^ band at apical third, slightly curving forward as a caret character, not reaching suture. Legs reddish brown, thinly clothed with suberect pale yellow hairs, moderately elongated; femora moderately clavate; hind femora nearly reaching but not exceeding elytral apices; first meta-tarsomere longer than total length of remaining tarsomeres and at least twice as long as 2^nd^ and 3^rd^ combined. Sternites finely closely punctate, clothed with yellow pubescence except for the basal margin of the 2^nd^ sternite.

Female (Fig. 1b, d, f): Mostly as the male except for: body somewhat larger on average; 7^th^ antennomere surpassing the humeri; 1^st^ elytral band wider, sutural vertex reaching suture and scutellum stripe for some individuals; 3^rd^ elytral band wider sometimes, reaching suture or connecting to the 2^nd^ marking in some individuals; meta-femora not reaching the elytral apex; yellow hairs not covering the basal margins of the 3^rd^, 4^th^ and 5^th^ sternite. Length 14.2–17.8 mm.

#### Etymology.

The name of the new species was inspired by the pensive face emoji-like pattern formed by the transverse black stripes on its elytra.

### Modified couplets to key by Gressitt (1951) of Chinese *Xylotrechus* species group above to accommodate the new species

**Table:** 

24	Pronotum with a distinct median black stripe	**25**
–	Pronotum lacking a distinct median black stripe, but with several indistinct blackish areas on disk	***savioi***
25	Pronotum with lateral discal spots more or less separated, not joined to basal band by curved lateral stripes	**26**
–	Pronotum with median stripe joining a subbasal band that curves forward to join lateral spots of disc	***polyzonus***
26	Frons with a single carina	**27**
–	Frons with a V-shaped carina or striated projected stripe	**30**
27	Four transverse bands on each elytron, the first maybe reduced to a marking in the middle of elytron; the hind two curved toward base, or the third one curved toward base and the last broken into two markings	**28**
–	Three transverse black bands on each elytron, the last covers apical third of elytron	***klapperichi***
28	Second elytral band widely separated from third band	***incurvatus incurvatus***
–	Second elytral band curving backward on disc nearly to third band	**29**
29	Last elytral band not broken into two markings	***incurvatus contortus***
–	Last elytral band broken into two markings	***atrolineatus***
30	Elytra with ground color entirely black, clothed with yellow pubescence and with a common postsutural spot and each with three longitudinal stripes of black	***multisignatus***
–	Elytra with only two post-scutellum longitudinal short stripes, no other longitudinal stripes	***tristisfacies* sp. n.**

## Supplementary Material

XML Treatment for Xylotrechus tristisfacies
